# Cold adaptation and horizontal gene transfer shape Antarctic sponge microbiomes

**DOI:** 10.1186/s40168-025-02262-z

**Published:** 2025-11-26

**Authors:** Maria F. Manrique-de-la-Cuba, Marileyxis López-Rodríguez, Sebastián Abades, Nicole Trefault

**Affiliations:** 1 GEMA Center for Genomics, Ecology & Environment, Universidad Mayor, Santiago, Chile; 2 Millennium Nucleus in Marine Agronomy of Seaweed Holobionts (MASH), Puerto Montt, Chile; 3 FONDAP Center IDEAL — Dynamics of High Latitude Marine Ecosystem, Punta Arenas, Chile

**Keywords:** Sponge microbiome, Antarctica, Metagenomics, MAG, Cold, Adaptation, Horizontal gene transfer, Sponge symbiont

## Abstract

**Background:**

Marine sponges exhibit wide distribution in tropical, temperate, and polar environments. They host diverse microbiomes important to their survival and ecological roles. Antarctic sponges, thriving in extreme cold environments, harbor unique microbial communities. However, functional differences distinguishing Antarctic sponge microbiomes have been poorly investigated. In this study, we investigated how the functional composition of the microbiomes of Antarctic sponges differs from that of their counterparts in other environments, with a particular focus on functions related to cold adaptation. We also assessed the role of horizontal gene transfer (HGT) in driving these functional adaptations.

**Results:**

Antarctic sponge microbiomes displayed a unique functional signature characterized by significantly higher proportions of genes related to cold adaptation, such as cold shock proteins, chaperones, heat shock proteins, and osmoprotectants, compared to their tropical and temperate counterparts, and antioxidants compared to the surrounding seawater. HGT was prevalent in Antarctic sponge symbionts, particularly in the dominant Gammaproteobacteria, Alphaproteobacteria, and Bacteroidia, contributing equally to metabolic functions and cold adaptation, with an important fraction of the latter exhibiting long-distance horizontal gene transfer (HGT). Conjugation, primarily mediated by integrative and conjugative elements (ICE), is a proposed crucial mechanism driving horizontal gene transfer (HGT) in Antarctic sponge symbionts. The cold shock protein C (CspC), linked to cold adaptation, was restricted to Proteobacteria and identified as a potential horizontally acquired gene exclusive to sponge symbionts compared to free-living bacteria in the Antarctic marine ecosystem.

**Conclusions:**

Antarctic sponge microbiomes exhibit higher proportions of functional adaptations for cold environments facilitated by horizontal gene transfer (HGT). These findings highlight the evolutionary importance of HGT mechanisms in shaping microbial symbioses in extreme environments. Further exploration of HGT dynamics and the role of specific symbionts in cold adaptation could reveal novel insights into microbial evolution and host–symbiont interactions in polar ecosystems.

Video Abstract

**Supplementary Information:**

The online version contains supplementary material available at 10.1186/s40168-025-02262-z.

## Background

Sponges are found in temperate, tropical, and polar regions, occupying benthic habitats from shallow coastal waters to deep-sea ecosystems [1, 2]. They significantly contribute to carbon, nitrogen, and silicon cycling by filtering large volumes of seawater and transforming dissolved organic matter (DOM) into forms accessible to other organisms, thereby supporting benthic food webs [2, 3]. Other ecological roles of sponges include habitat provision and symbiotic relationships with microorganisms from the three domains of life that enhance nutrient cycling [2].

As in other animal–microbe symbioses, the sponge microbiome is essential for maintaining the host’s health and development [4–6]. Symbionts provide essential functions to their hosts, such as nutrients (e.g., vitamin synthesis) [7] and defense and immune support [8–10]. The bacterial and archaeal communities associated with marine sponges are variable in composition and diversity depending primarily on the host phylogeny [11–18], bacterial abundance [5, 14, 19–22], biogeography or location of the host [11, 14, 23], and environmental factors [24–26]. However, despite extensive research on microbial community composition, the functional variability of the sponge microbiome across different hosts and environmental conditions remains largely unexplored.

Antarctic sponges make up a significant portion of the occupied living benthos [27, 28] and have the greatest influence on the dynamics and structure of this ecosystem [29]. The bacterial and archaeal microbiomes of Antarctic sponges are distinct in both diversity and composition compared to those of tropical and temperate counterparts [11, 30, 31]. Habitat-specific and habitat-generalist bacteria are associated with Antarctic sponges [11]; however, whether the functions encoded in their symbiont genomes also differentiate the Antarctic sponge microbiomes remains an open question.

Sponge microbiomes differ significantly in composition and specific functions from those of the surrounding seawater [10, 15, 32, 33], and this also holds for Antarctica [31, 34, 35]. Sponge symbionts are commonly enriched in functions related to the symbiotic lifestyle, such as CRISPR systems, restriction modification systems, eukaryotic-like proteins, mobile genetic elements (MGE), vitamin synthesis, secondary metabolism and bioactive compounds, and attachment to host tissues (e.g., fibronectins and cadherins) [10, 33, 34].

MGE include plasmids, transposons, integrons, phages, and integrative and conjugative elements (ICE), which facilitate horizontal gene transfer (HGT) and the exchange of genetic material between species that are not parentally related [36–38]. HGT is a major contributor to microbial evolution, especially for bacteria, but is less prevalent among eukaryotes [37, 38]. This process can provide bacteria and archaea with new traits, such as antibiotic resistance and metabolic capabilities, enabling bacterial survival, species diversification, and niche expansion [36, 37]. HGT can involve the adaptation of specific symbionts to a common host, facilitating functional convergence [33]. For instance, it potentially expanded the heterotrophic metabolism of the most prevalent Gammaproteobacteria symbiont “AqS1” in the sponge *Amphimedon queenslandica* from Alphaproteobacteria donors [39]. Likewise, there is evidence that the symbiont *Nesterenkonia* sp., isolated from the Antarctic sponge *Iophon* sp*.,* has horizontally acquired the vitamin B5 synthesis-related gene *panE* from Actinobacteria members [40]. However, whether other functions in Antarctic sponge symbionts are acquired through HGT remains unaddressed.

Antarctic habitats are reservoirs of psychrophilic and psychrotolerant microorganisms [41, 42]. They have evolved functional adaptations to thrive in cold environments while maintaining their cell integrity, enzyme kinetics, membrane fluidity, osmotic pressure, water viscosity, and molecular interactions [43]. HGT has been proposed to participate in acquiring functions for cold adaptation in free-living microorganisms [44–47]. Plasmids in psychrophilic and psychrotolerant bacteria often carry genes related to cold adaptation, including those involved in protecting against cold-induced stress, scavenging reactive oxygen species, and modifying membrane fluidity [46]. Although functions for cold adaptation in sponge microbiomes from Antarctica have been reported previously [35], their proportions and distribution are not well understood yet. Moreover, the contribution of HGT in acquiring these functions remains unexplored.

Here, we aimed to explore functional differences in the microbiomes of Antarctic sponges compared to those of (i) tropical and temperate sponges and (ii) their surrounding seawater, with a particular focus on functions related to cold adaptation and the contribution of HGT in acquiring these traits. Our findings revealed that while the general functional microbiome of sponges was broadly conserved across environments, Antarctic sponge microbiomes exhibited distinct proportions of cold-adaptation-related functions, including cold shock proteins, chaperones, osmoprotectants, heat shock proteins, and antioxidants. Moreover, potential HGT events were more frequent in Antarctic sponge symbionts than in free-living seawater bacteria. Interestingly, HGT contributed equally to cold adaptation and metabolic functions of known high transfer rate. The *csp*C gene, which was restricted to Proteobacteria but present in all the Antarctic sponge species analyzed, is a potential candidate for horizontal acquisition in sponge symbionts but not in free-living microorganisms of the surrounding Antarctic seawater.

## Methods

### Antarctic sample collection

Adult sponge samples (*n* = 9) were collected during three expeditions between 2018 and 2021 from Chile Bay, Greenwich Island, South Shetland Islands, Antarctica, at depths ranging from 11 to 17 m (Supplementary Table S1). Sponge samples were collected by scuba diving and kept individually in plastic bags containing natural seawater until the initial processing at the laboratory in Captain Arturo Prat Base at Greenwich Island.

Seawater samples (*n* = 4) were collected approximately 5 m from each sponge sampling site using a 5-L Niskin bottle (Supplementary Table S1). The samples were prefiltered on board through a 150-μm pore mesh to remove large particles, stored in an acid-washed carboy, and kept in the dark until processing in the laboratory.

### Sponge identification

The external morphology of the sponges was first examined using sponge samples and field images of specimens collected during expeditions, considering color, general shape, consistency, and surface features. For taxonomic identification, skeletal components (e.g., spicules and spongin fibers) were examined under a stereo microscope and a light microscope as described in detail by Hajdu et al. (2011) [48]. For this, subsamples of sponge tissues previously fixed in 100% ethanol were treated in sodium hypochlorite (commercial bleach) in distilled water and incubated at 70 °C for 30 min to remove organic material prior to microscopic observation. For sponge taxa containing spicules, the bleach mixture was prepared at a 1:1 ratio. The released spicules were thoroughly rinsed with distilled water. Sponges identified by this procedure included *Myxilla* (*Burtonanchora*) *lissostyla* (with smooth styles and spined heads), *Mycale acerata* (with long, thin oxeas and lacking echinating acanthostylos or anisochelae), and *Iophon* sp. (with tylostyles and distinctive microscleres). For sponges lacking spicules, the mixture was prepared at a 1:3 ratio. The resulting spongin fibers were rinsed with distilled water, and the analysis consisted of examining the spongin network architecture under both a stereo microscope and a light microscope for species-level identification. The only sponge species identified by this procedure was *Dendrilla antarctica* (with a regular and tree-like network of spongin fibers with repeated branching).

### Sample treatment, DNA extraction, and sequencing

Sponge samples were rinsed three times with sterilized seawater, cleaned under a stereomicroscope to remove dirt and ectoparasites, and stored at −80 °C until further processing in Santiago, Chile. Subsamples of about 1 cm^3^ of each sponge tissue were obtained with a sterile scalpel blade and rinsed again to remove loosely attached microorganisms from seawater. The subsamples were processed according to Rodríguez-Marconi et al. (2015) [31], which include tissue disruption, filtration, centrifugation, and separation of the microbial fraction.

Seawater samples were filtered through 20-μm (NY20), 3-μm (GSWP), and 0.2-μm (GPWP) pore size filters of 47 mm in diameter (Millipore) using a Swinnex holder system and a Cole-Parmer 1–600 rpm peristaltic pump. Filters were stored in 2-mL cryovials at −20 °C until DNA extraction.

After the sponge treatment, the metagenomic DNA in the resulting pellets was extracted using the DNeasy PowerSoil Pro Kit (QIAGEN) according to the manufacturer’s instructions. DNA extraction from the seawater samples was performed using the N-cetyl N,N,N trimethylammonium bromide (CTAB)-based protocol described by Rodriguez-Marconi et al. (2015) [31]. Briefly, filters were thawed, and half were cut into small pieces and treated with a CTAB extraction buffer (10% CTAB, 0.7% NaCl) to recover DNA.

The quantity and integrity of the DNA extracted in each case were evaluated with a Qubit fluorometer and by 0.8% agarose gel electrophoresis, respectively, and stored at −20 °C until further steps. Shotgun metagenomic sequencing was performed by Genoma Mayor (https://www.genomamayor.com) with the Illumina HiSeq platform (150 bp paired-end) (Supplementary Table S1). The library preparation and sequencing followed the protocol initially described by Comeau et al. [49] and subsequently updated by Comeau and Filloramo [50].

### Sponge metagenome dataset collection

We retrieved raw paired-end metagenomic reads from Antarctic sponge microbiomes (*n* = 8) available in the SRA database (Supplementary Table S1**)**. This dataset belongs to sponges of several species sampled between 2013 and 2015 from Fildes Bay, King George Island, South Shetland Islands, Antarctica.

We also retrieved all publicly available Illumina shotgun-sequenced metagenomes until June 2022, derived from sponge microbiomes of tropical (*n* = 15) and temperate (*n* = 13) environments, stored as raw reads in the SRA database (Supplementary Table S1). Three metagenomes correspond to deep-sea sponges of the species *Neamphius huxleyi,* *Hymedesmia (Stylopus) methanophila*, and *Iophon methanophila* (IDs SRR496756, SRR7464845, and SRR7464866, respectively). These metagenomes belong to sponges of several Demospongiae species sampled from 2003 to 2018 across several locations. A cyanobacterial trichome-enriched metagenome, though meeting the previous criterion, was not included in the processing.

### Metagenome pre-processing and assembly

The quality control of all raw metagenomic reads from sponge and seawater microbiomes was checked using FastQC v0.11.9 [51] and MultiQC v1.10.1 [52]. TruSeq and Nextera adapters were removed with Skewer v0.2.2 [53] (Supplementary Table S1). The parameters were fixed as -r = 0.03, -k = 16, and -l = 50 for TruSeq adapters and -r = 0.05, -k = 9, and -l = 50 for Nextera adapters and the rest by default. The trimmed reads with average quality below 28 were discarded using BBDuk from BBTools v39.01 [54]. For seawater microbiomes, we joined raw reads from different size fractions (i.e., 0.2–3 µm, 3–20 µm, and 20–150 µm) of the same sample to assemble whole microbiomes (*n* = 4, fractions detailed in Supplementary Table S1). For assembling the quality-filtered reads to contigs, MEGAHIT v1.2.9 [55] was used with read pairs and default parameters. Then, the quality of the assemblies was assessed using Quast v5.2.0 [56] (Supplementary Table S2).

### Metagenome functional annotation and filtering

Gene prediction from contigs was performed by requesting nucleotide and amino acid sequences with the metagenomic procedure of Prodigal v2.6.3 [57]. Functional annotation was carried out using eggNOG-mapper v2.1.11 [58], aligning the amino acid sequences with Diamond v2.1.6 against the eggNOG database v5.0 [59]. Gene Ontology (GO) terms inferred from electronic curation were omitted *to prevent increased risk of false positives and ambiguous functional assignments, as genetic context from mixed communities is highly fragmented*. Seed ortholog IDs assigned by eggNOG were extracted separately for each environment to compare exclusive and shared genes using the ggVennDiagram package v1.5.2 [60] in R Statistical Software v4.2.1 (R Core Team 2023) [61].

Functions for cold adaptation were defined and classified into eight categories: antifreeze proteins (four nonredundant-related genes), antioxidants (1561 nonredundant-related genes), chaperones (8325 nonredundant-related genes), cold shock proteins (Csp, 1,059 nonredundant-related genes), fatty acid desaturases (1294 nonredundant-related genes), heat shock proteins (Hsp, 3405 nonredundant redundant genes), nucleotide repair proteins (16,638 nonredundant related genes), and osmoprotectants (869 nonredundant related genes). An in-house bash script was developed to search for and count keywords associated with each category of cold adaptation functions across non-eukaryotic metagenome annotations. The filtered annotations were manually checked to eliminate functions incorrectly related by keyword coincidence during the final script execution. The list of nonredundant cold adaptation genes is provided in Supplementary Table S3.

Functions related to amino acid transport and metabolism (ATM, 182,806 nonredundant genes), carbohydrate transport and metabolism (CTM, 107,476 nonredundant genes), energy production and conversion (EPC, 132,594 nonredundant genes), and lipid transport and metabolism (LTM, 88,393 nonredundant genes) were identified by classifying genes annotated in these respective Cluster of Orthologous Groups (COG) categories using the same in-house script. Functions related to metal and antibiotic resistance and metabolism (MAR, 20,427 nonredundant genes) and the machinery of integrative and conjugative elements (ICE, 4651 nonredundant genes) were similarly identified through keyword searches and counting. The filtered annotations were then manually curated to remove incorrectly assigned functions based on keyword matches in the final script execution. The list of nonredundant metabolic, resistance, and conjugation genes is provided in Supplementary Table S4.

Gene counts of all filtered functions (cold adaptation, metabolism, resistance, and conjugation) were converted to percentages over the total genes annotated in each metagenome. Statistical pairwise comparisons between metagenomes of different environments/habitats were performed using *t*-tests in the ggpubr package v2.6–4 of R [62].

For the presence/absence analysis of genes for cold adaptation, sequence subsampling of the quality-filtered reads was carried out to equal the sample with the lowest sequence number (ID SRR7783610, *Cinachyrella australiensis;* 266,595 sequences) using seqtk v1.2 [63] during the metagenome pre-processing at the beginning. Then, assembly, gene prediction, and functional annotation were performed following the steps previously detailed for non-subsampled metagenomes. Hierarchical clustering in the heatmap was performed using Euclidean distances and Ward’s method with the ComplexHeatmap package v2.19.0 [64] in R and seed set to 1000.

Exclusive Csp genes in sponge microbiomes of each environment were calculated by extracting per-environment seed ortholog IDs assigned by eggNOG and using ggVennDiagram and the *setdiff* function (base package) in R. Then, the occurrence counts of exclusive per-environment genes were measured using the in-house script and manual conversions of counts to percentages.

### Gene abundance normalization

For gene abundance calculation, the quality-filtered reads were mapped against each metagenome nucleotide sequence of predicted genes using Bowtie 2 v2.5.1 [65]. In this execution, the built index was forced to be “large” with a seed equal to 1000, and the very sensitive mode was applied. The sequence alignment map (SAM) output order was set to match the order of the input reads. Then, the pileup function of BBmap v39.01 [66] was used to generate gene coverage information from SAM files as reads per kilobase per million mapped reads (RPKM). The average genome size (AGS) was estimated for gene abundance normalization by aligning universal single-copy genes to metagenomes using MicrobeCensus v1.1.1 [67]. Reads per kb per genome equivalent (RPKG) were calculated using the number of reads mapped to each gene, the gene length (kb), and the genome equivalents of each metagenome, as output information supplied by Pileup and MicrobeCensus.

For functional composition analysis, we employed two complementary comparison approaches: (i) at the COG major category level, which is less sensitive to noise, and (ii) at the individual gene level, which provides fine-scale resolution. To identify trends or differences between environments at the major category level, RPKG values of non-eukaryotic genes were summed within the same COG categories—as assigned based on eggNOG annotations. Genes without COG annotations were classified as “unassigned” and summed separately. Relative abundances of COG categories were visualized using stacked barplots. To detect environmentally related differences in gene presence, absence, or abundance, at the individual gene level, all non-eukaryotic RPKG values were extracted to create an abundance matrix with seed ortholog IDs assigned by eggNOG as rows and metagenome IDs as columns, using the fossil package v0.4.0 in R [68]. Ordination analysis was performed on this matrix using Bray–Curtis distances in three dimensions with the Vegan package v2.6–4 [69]. Statistical comparisons of between-gene distances were conducted using analysis of similarity (ANOSIM, anosim function in Vegan) and non-parametric multivariate analysis of variance (PERMANOVA, adonis2 function in Vegan, permutation = 999, sed.seed = 1000).

Pairwise comparisons between metagenomes from different environments and habitats were also conducted based on the gene abundance and diversity of functions related to cold adaptation, metabolism, resistance, and conjugation, which were previously analyzed. Non-eukaryotic RPKG and Shannon indexes for each functional category were extracted, summed, and compared using search patterns described before (in-house script) and *t*-tests in R. Shannon index calculation followed the formula reviewed by Ortiz-Burgos (2016) [70].

### Metagenome binning

Only the Antarctic metagenomes from sponges (*n* = 9) and seawater (*n* = 4) were processed, corresponding to the Greenwich Island sampling. Binning of contigs was performed using metaWRAP [71] v1.3.2, running the MaxBin [72] and metaBAT [73] algorithms at the same time for each metagenome. The refinement module of metaWRAP consolidated the bins produced by the two algorithms in a single bin set. In this step, we recovered high-quality bins only, defined by a minimum of 70% of completeness and a maximum of 5% of contamination. These parameters were verified using CheckM [74] in the same pipeline. We discarded one sponge metagenome (ID GM2034-3) for lacking bins that passed the thresholds after the refinement. High-quality bin abundances in each sample, expressed as genome copies per million reads, were calculated by providing the entire non-binned assembly in the quantification module of MetaWRAP. Afterward, the re-assemble bins module was used to improve the final bin set separately with a “permissive” and “strict” algorithm. Only the bins that improved through reassembly were altered in the final set, and the rest remained the same. In our study, these final bins were named metagenome-assembled genomes (MAGs).

### Phylogenomic inference and functional annotation of MAGs

MAG taxonomy assignment was made separately for MAGs from Antarctic sponges and Antarctic seawater using the “classify workflow” of GTDB-Tk v2.3.2 [75] in two executions. The first run included the Mash reference sketch database, while the second run skipped the ani_screening step to classify genomes using Mash and Skani. Summary reports produced by both runs were equal. Skipping the ANI screening provided multiple sequence alignments (MSA) that included all MAGs, not only those without an ANI reference. The MSA of the user-provided MAGs from Antarctic sponges and seawater was used as an input in the IQ-TREE web server [76] to infer phylogenomic trees under the maximum likelihood method. We used autodetection of the suitable substitution model through Bayesian information criterion (BIC), 1000 bootstrap alignments, 1000 iterations, and other parameters by default. Visualization and customization of the resulting bacterial and archaeal trees were managed in the iTOL web server [77] with GTDB taxonomy assignments.

The functional annotation of MAGs was carried out similarly to metagenomes using Prodigal, eggNOG-mapper, and the EggNOG database but requesting all GO evidence available to maximize annotation coverage (beneficial for poorly studied lineages) and to leverage the higher completeness and organization of MAGs compared to metagenomes. Functional categories related to cold adaptation, metabolism, resistance, and conjugation were extracted using the same in-house scripts and steps as described previously for metagenomes.

### HGT detection in MAGs

Prediction of horizontally transferred (HT) genes (i.e., horizontally acquired genes) in MAGs from Antarctic sponges and seawater was performed with HGTector2 v2.0b3 [78]. The Diamond aligner was selected with default parameters in the batch homology searching on the predicted protein sequences against NCBI RefSeq genomes without applying taxonomic filters. HGT prediction (search and analyze commands) followed default parameters but reported the taxon names of the potential donors.

The annotations of the predicted HT genes were extracted and counted, especially for the functions related to cold adaptation, metabolism, resistance, and conjugation analyzed before, following the in-house scripts. Percentages of HT genes of an interest function “X” were calculated in two ways: (i) Number of HT genes of the function “X” over the number of total HT genes in each MAG and (ii) number of HT genes of the function “X” over the total genes annotated in each MAG. Correlation between these two metrics and the percentage of genes annotated in each function “X” was measured by Pearson’s method using the ggpubr package in R. Wilcoxon tests were used to compare HT genes of the functional groups analyzed between MAGs from Antarctic sponges and seawater. Additionally, annotations of characterized Csp and antioxidants were extracted to track their distribution, proportion, and horizontal acquisition, contrasting MAGs from Antarctic sponges and seawater through the ggalluvial package v0.12.5 in R [79]. Uncharacterized genes were omitted from this analysis.

To detect community-level HT genes in sponge symbionts from seawater free-living donors, MetaCHIP v1.10.13 [80] was performed between groups (sponges vs. seawater) and among taxonomy levels in two independent executions. We retrieved all putative recipient genes detected in sponges for both executions and discarded those coming from symbiont donors. These genes were annotated as described before for MAGs.

## Result

### The microbiomes of Antarctic, tropical, and temperate sponges exhibit typical and similar broad functional profiles

**Fig. 1:**
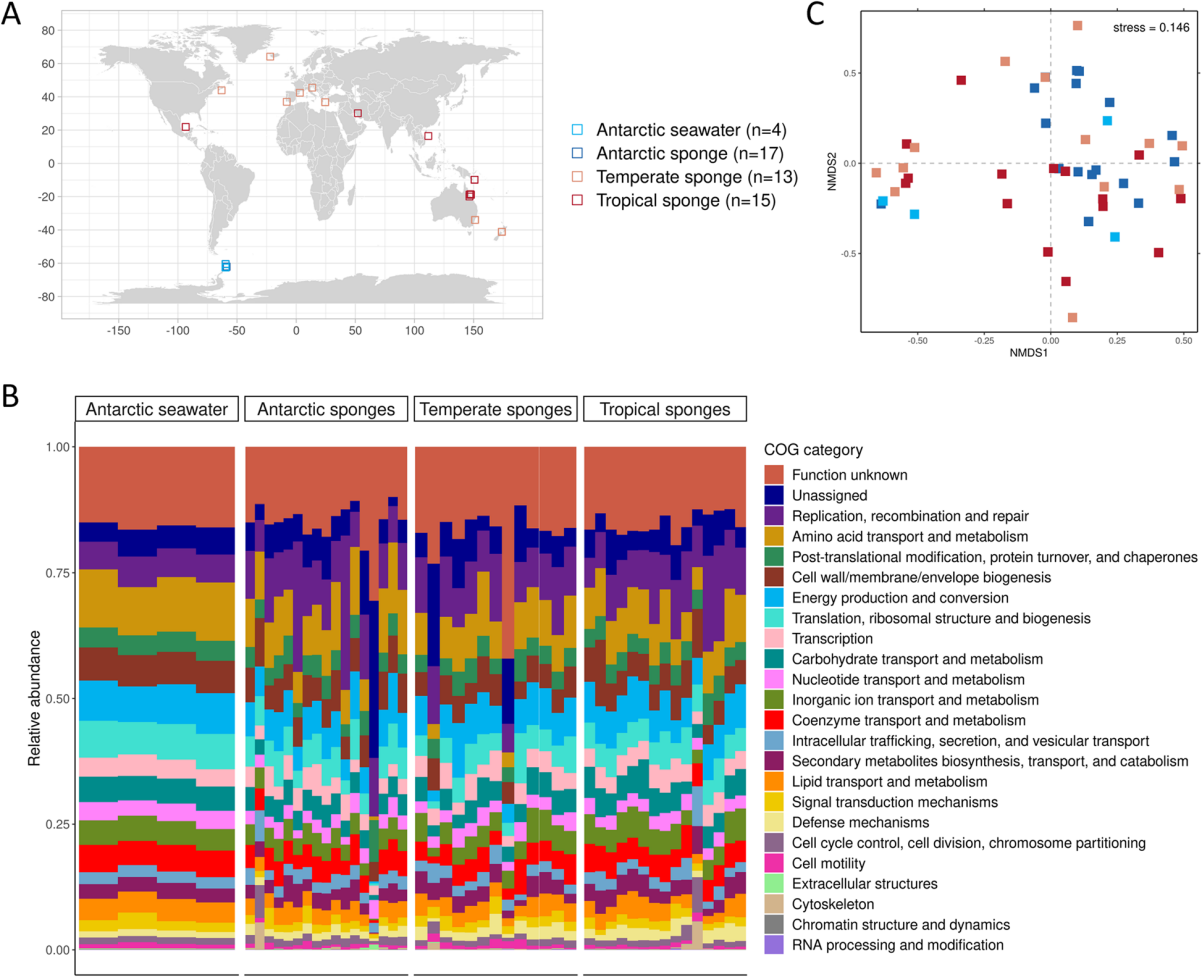
Geographic distribution of the samples used in this study and functional profiles of their microbiomes. **A** Geographic distribution of the sponge and seawater samples. **B** Functional composition based on COG categories contrasting the sponge microbiomes across different environments and the Antarctic seawater. **C** nMDS of gene composition based on Bray–Curtis dissimilarities

We explored the functions of 45 sponge microbiomes from tropical, temperate, and Antarctic marine environments (Fig. 1A and Supplementary Table S1). We also included four microbiomes from the surrounding seawater of the Antarctic sponges. The sponges belonged to several orders within the class Demospongiae and one genus within the class Calcarea. We recovered metagenome sizes between 2.73 and 869 Mb, encompassing 6305 to 1,318,174 predicted genes (Supplementary Table S2). From them, we functionally annotated 2668 to 1,041,343 non-eukaryotic genes. Our exploration compared the sponge microbiomes by environment and the Antarctic microbiomes by habitat (i.e., sponge and seawater).

We tested whether the sponge microbiomes differed in their functional profiles by grouping the genes into categories of Clusters of Orthologous Groups (COG) to identify major functional trends between environments (Fig. 1B). The functional categories “Replication, recombination, and repair” and “Amino acid transport and metabolism” were the most abundant in the sponge microbiomes of the three environments, while the categories “RNA processing and modification,” “Chromatin structure and dynamics,” and “Cytoskeleton” showed the lowest abundances. COG containing symbiotic functions were more enriched on average in microbiomes from sponges than seawater: “Replication, recombination, and repair” (3445 RPKG vs. 548 RPKG), “Defense mechanisms” (437 RPKG vs. 122 RPKG), “Post-translational modification, protein turnover, and chaperones” (1651 RPKG vs. 386 RPKG), and “Secondary metabolites biosynthesis, transport, and catabolism” (687 RPKG vs. 283 RPKG), which often include restriction-modification systems, CRISPR systems, eukaryotic-like proteins, and bioactive compounds, respectively.

We also performed a gene-level comparison to detect environmentally related differences in the presence, absence, or abundance of specific genes. However, the sponge microbiomes exhibited very weak differentiation in their functional composition, either between or within environments, based on Bray–Curtis dissimilarities at the gene level (ANOSIM, *p* = 0.008, *R* = 0.1025), displaying a dispersed pattern in ordination (Fig. 1C). Significant differences emerged between the microbiomes of tropical and Antarctic sponges but explained only a minimal variation in the functional composition (pairwise PERMANOVA, *p*-adjusted = 0.024, *r* = 0.0482, Supplementary Table S5). Additionally, the sponge microbiomes of different environments shared very few genes (Supplementary Fig. S1), indicating that the microbial genes in sponges perform similar or equivalent functions but correspond to unique orthologs (i.e., gene representatives from different species).

### Functions for cold adaptation differentiate the microbiomes of Antarctic sponges

We explored functions related to cold adaptation in the sponge and seawater microbiomes, including genes encoding and regulating antifreeze proteins, antioxidants, chaperones, Csp, fatty acid desaturases, Hsp, nucleotide repair proteins, and osmoprotectant-related pathways. The *patterns of presence and absence for* genes within these functions were evenly distributed among tropical, temperate, and Antarctic microbiomes, displaying no clustering by environment or Antarctic habitat after sequence subsampling to equalize sequencing depth across samples (Supplementary Fig. S2). Notably, antifreeze genes were exceedingly rare — only four were detected: one in the microbiome of the tropical sponge *Coscinoderma mathewsi* and three in Antarctic seawater microbiomes — and were not retained in the analysis after sequence subsampling (see “Methods”).

**Fig. 2:**
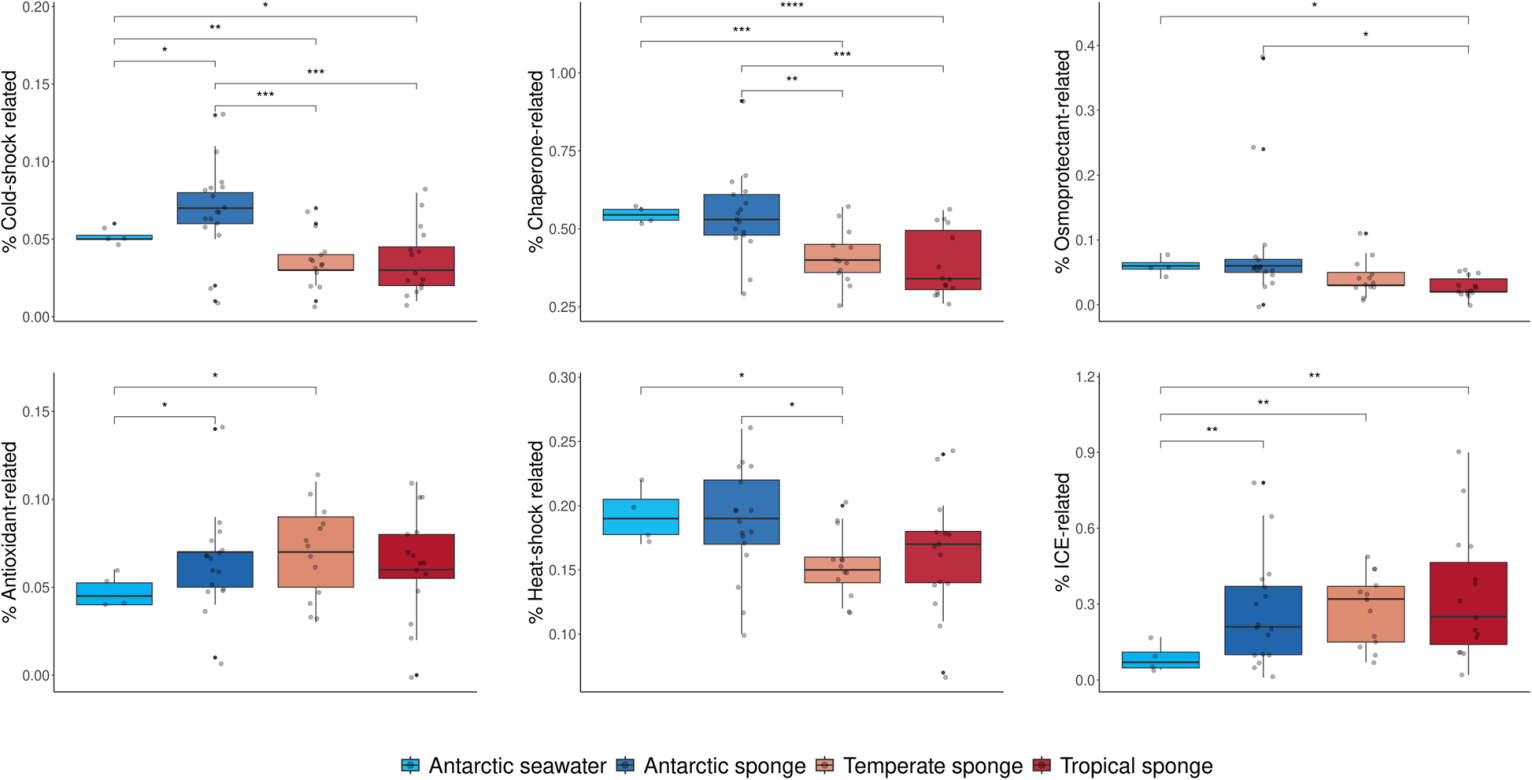
Percentage of genes encoding functions related to cold adaptation and conjugation significantly higher in Antarctic sponge microbiomes compared to their surrounding seawater and temperate and tropical sponge microbiomes. ****p < 0.0001, ***p < 0.001, **p < 0.01, *p < 0.05. ICE, machinery of integrative and conjugative elements

We were interested in determining functions for cold adaptation from Antarctic sponge microbiomes that significantly exceed those from other environments in terms of representation percentage, which we called an “Antarctic sponge microbiome signature.” This signature was evident for functions related to Csp, chaperones, Hsp, and osmoprotectants through pairwise comparisons based on the proportion of annotated genes (Fig. 2, Supplementary Fig. S3, and Supplementary Table S6). However, three deep-sea tropical sponges also showed increased levels of these functions, most notably *N. huxleyi*, which showed similar values to the Antarctic pattern. *Hymedesmia (Stylopus) methanophila* showed high proportions of chaperones and Hsp, whereas *I. methanophila* exhibited elevated percentages of Csp. Thus, while Antarctic sponges presented the clearest overall signature, these deep-sea sponges converged on an Antarctic-like profile in specific functions.

Notably, Csp was the sole function linked to cold adaptation, with a higher mean percentage in Antarctic sponge microbiomes compared to both Antarctic seawater and sponges from tropical and temperate regions, excluding the deep-sea species noted above. Antarctic and tropical sponge microbiomes each contained around one-third of the total Csp-related genes unique to their respective environments (see Supplementary Fig. S4). Only one tropical sponge microbiome lacked *csp* genes. Most of the unique genes were shared among fewer than 50% of the sponges within the same environment. Nevertheless, one putative *csp* gene with unstandardized nomenclature (ID 313603.FB2170_01856) was found exclusively in 60% of the Antarctic sponges.

Antioxidants were the only function showing similar percentages among sponge microbiomes but at higher levels in Antarctic sponges compared to Antarctic seawater, which we labelled a “sponge microbiome signature” (Fig. 2). This latter signature extended to conjugative functions, which are often linked to a symbiotic lifestyle. Genes encoding the machinery ICE were significantly enriched in the sponge microbiomes from tropical, temperate, and Antarctic environments compared to the surrounding Antarctic seawater (Fig. 2).

Additionally, Antarctic sponge microbiomes presented significantly more RPKG for antioxidants and fatty acid desaturases compared to seawater and more osmoprotectant-related functions compared to tropical sponges (Supplementary Table S6). The diversity of functions related to cold adaptation, measured by the Shannon index, was instead higher in the Antarctic seawater microbiomes (Supplementary Table S6).

We also explored abundant and essential microbiome metabolic functions to contrast whether the Antarctic sponge signature expands beyond cold adaptation. For convenience for further analyses, we selected functions known to be present at high HGT rates, including amino acid transport and metabolism (ATM), carbohydrate transport and metabolism (CTM), energy production and conversion (EPC), lipid transport and metabolism (LTM), and metal and antibiotic resistance and metabolism (MAR). However, a signature of the Antarctic sponge microbiome was not evident for these functions (Supplementary Fig. S3 and Supplementary Table S6). In fact, Antarctic seawater exhibited significantly higher percentages of genes involved in ATM, CTM, LTM, and MAR compared to sponge microbiomes of the different environments (Supplementary Fig. S3).

### HGT has a high contribution to acquiring functions for cold adaptation in dominant Antarctic sponge symbionts

**Fig. 3:**
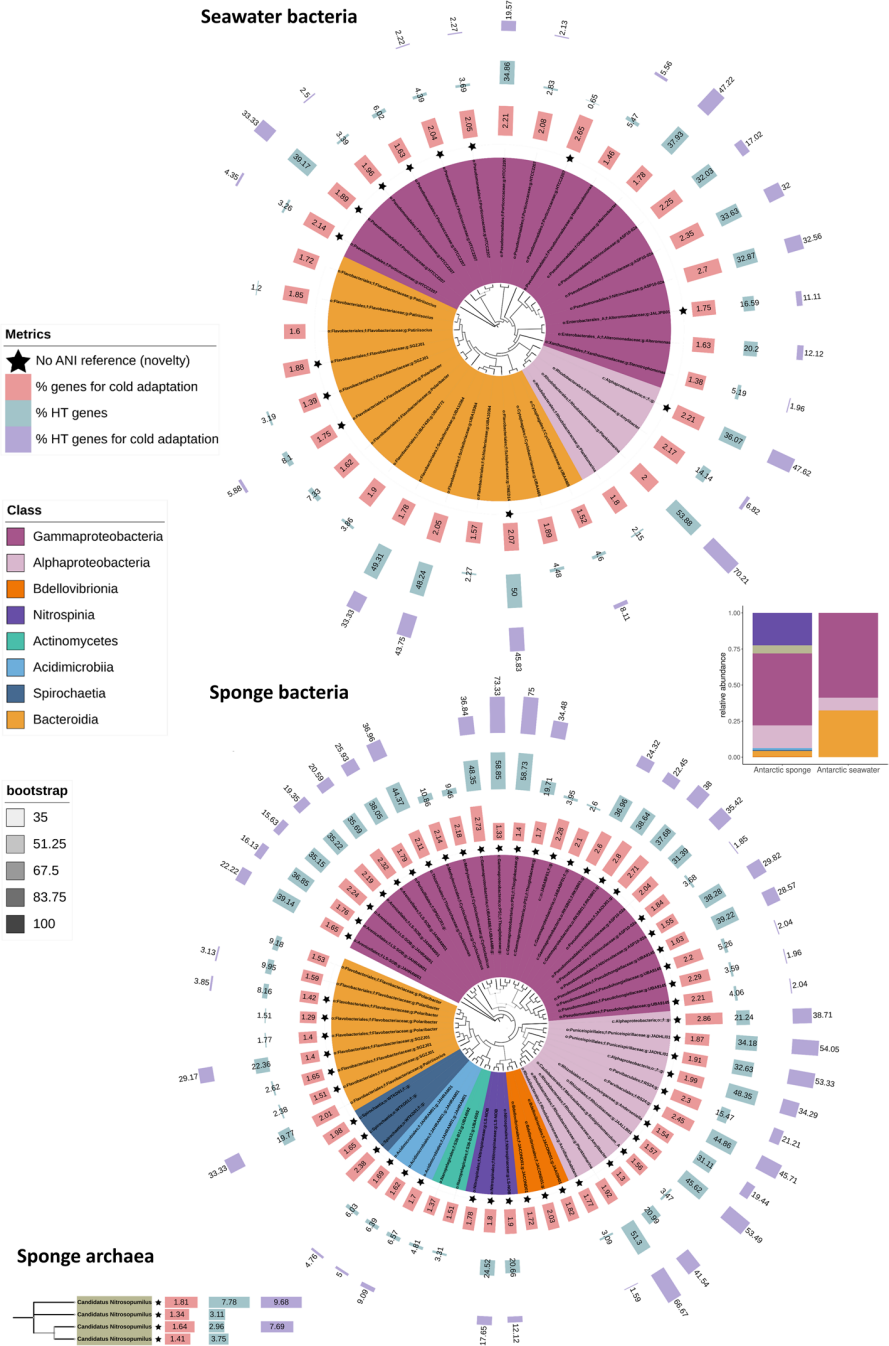
Phylogenomic trees of MAGs corresponding to Antarctic sponge symbionts and free-living microorganisms in Antarctic seawater. The trees were constructed using the maximum likelihood method and GTDB taxonomy. Black stars indicate that the taxonomy was not assigned from a reported ANI reference genome, representing genomic novelty; single bars (the last three outer rings) indicate percentages of genes and horizontally transferred (HT) genes. The bar lengths were adjusted by ring for visualization; therefore, they are not on the same scale across rings. The taxa colors represent affiliations to a microbial class. The branch color gradient indicates bootstrap support. The barplot on the right displays the relative abundance of the MAG classes in Antarctic sponges and seawater

We explored the bacterial and archaeal metagenome-assembled genomes (MAGs) found in the Antarctic microbiomes by analyzing only a subset of sponges (*n* = 9, four species) and seawater (*n* = 4) from the same Antarctic expeditions (Supplementary Table S1). We recovered 836 initial bins, leading to 64 high-quality MAGs from eight Antarctic sponges and 35 from seawater (Fig. 3 and Supplementary Table S7).

More bacterial classes appeared in Antarctic sponges (Gammaproteobacteria, Alphaproteobacteria, Bacteroidia, Bdellovibrionia, Nitrospinia, Actinomycetes, Acidimicrobiia, and Spirochaetia) than in seawater (Gammaproteobacteria, Bacteroidia, and Alphaproteobacteria). Gamma- and Alphaproteobacteria were abundant as both sponge symbionts and free-living bacteria. *Candidatus Nitrosopumilus* (class Nitroshosphaeria) was the unique archaeal species retrieved and, in this case, associated only with the Antarctic sponges *Myxilla* (*Burtonanchora*) *lissostyla* and *Iophon* sp. Similarly, Antarctic sponges exhibited more undescribed microbial species than seawater, with most lacking ANI references, underscoring the microbial novelty of these ancient animals and the need for more draft genomes in global databases.

We then assessed the potential genes acquired through horizontal transfers, focusing on genes that encode functions for cold adaptation in contrast to ATM, CTM, EPC, LTM, MAR, and ICE. The number of horizontally transferred (HT) genes within each functional group, relative to the total number of HT genes, correlated directly with the gene content of each functional group (Pearson, *r* = 0.74, *p* < 2.2e-16, Supplementary Fig. S5A). Metabolic functions exhibited a higher gene content, followed by cold adaptation, resistance, and conjugation at the end (Supplementary Fig. S5A). To mitigate the bias of gene content in each functional group over the percentage of HT genes, our comparisons were based on the number of HT genes per total gene content within each functional group (Pearson, *r* = 0.1, *p* = 0.01, Supplementary Fig. S5B).

Gamma- and Alphaproteobacteria exhibited more putative HT genes than other taxa. Notably, along with Bacteroidia, they presented more HT genes as sponge symbionts than free-living microorganisms (Wilcoxon test, *p* < 0.05, Fig. 3). On average, HT genes accounted for 29% of the annotated genes in symbiotic Proteobacteria and 19% of the annotated genes in their free-living counterparts. The percentage of HT genes for cold adaptation was also higher in these bacterial classes but comparable between Antarctic sponges and seawater (Wilcoxon test, *p* > 0.05). When comparing all symbiont taxa with free-living bacteria, the differences were not significant (Wilcoxon test, *p* > 0.05). All symbiont and free-living taxa exhibited similar gene abundance for cold adaptation. However, MAGs assigned to the Bdellovibrionia and Spirochaetia classes, associated with Antarctic sponges, were the only ones lacking HT genes for cold adaptation (Fig. 3).

**Fig. 4:**
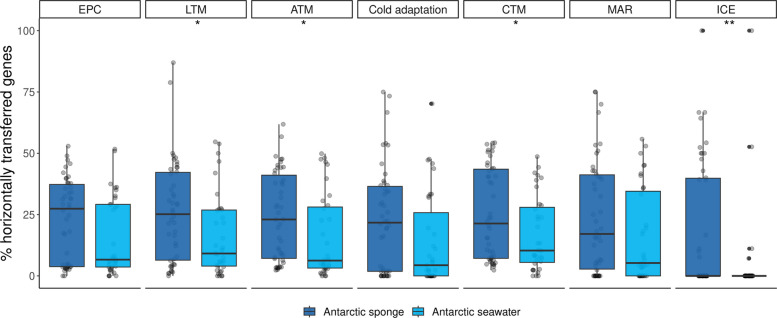
Percentage of putative horizontally transferred (HT) genes in Gamma- and Alphaproteobacteria and Bacteroidia, contrasting functional groups and Antarctic habitats (sponge vs. seawater). ATM, amino acid transport and metabolism; CTM, carbohydrate transport and metabolism; EPC, energy production and conversion; LTM, lipid transport and metabolism; MAR, metal and antibiotic resistance and metabolism; ICE, machinery of integrative and conjugative elements. Wilcoxon test, **p < 0.01, *p < 0.05

We also examined the role of horizontal gene transfer (HGT) in acquiring functions for cold adaptation by focusing on the dominant and HGT-proficient Gamma- and Alphaproteobacteria and Bacteroidia Antarctic sponge symbionts. The HGT contribution was measured by comparing the percentage of HT genes per each functional group used previously. The genes associated with cold adaptation showed similar percentages of horizontal transfers compared to those of metabolic and resistance functions analyzed, indicating a comparable contribution of HGT to the proportion or frequency of gene acquisition in these symbiotic bacteria (Fig. 4). In contrast, HGT contributed weakly to cold adaptation in free-living Gamma- and Alphaproteobacteria and Bacteroidia, primarily influencing CTM. Additionally, HGT for ATM, CTM, and LTM was significantly higher in these sponge symbionts than in their free-living counterparts (Fig. 4). Notably, we observed significantly more ICE that were HT in these sponge symbionts (13% more on average); such a difference remained significant when considering all taxa from Antarctic sponges against seawater, although less pronounced (Wilcoxon test, p < 0.05).

**Fig. 5:**
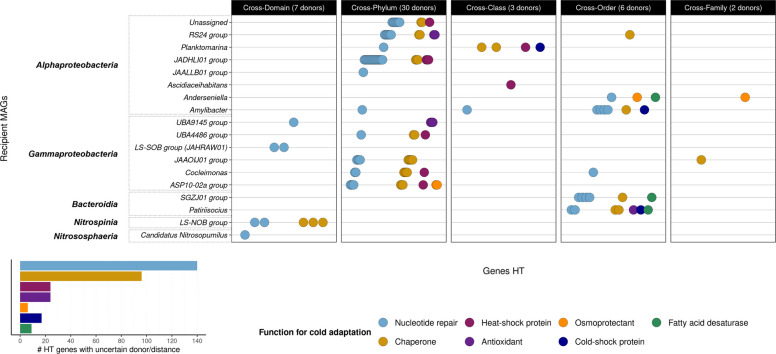
Taxonomic distance of the predicted HGT and proportion of horizontally transferred (HT) genes for cold adaptation in Antarctic sponge symbionts (recipient MAGs). The dot plot represents HT genes, with donor taxa assigned at least to the phylum level and with an identifiable taxonomic distance of transfer. Each dot corresponds to one HT gene; *X*-axis positions are arbitrary and used only to prevent dot overlap. The accompanying bar plot depicts the proportion of HT genes with unassigned donor taxa or lacking an identifiable taxonomic distance

Twenty-eight percent of the HT genes for cold adaptation in sponge symbionts came from donor taxa assigned at least at the phylum level (mainly the classes Actinomycetes, Gammaproteobacteria, and Bacteroidia) and reflecting potential long-distance HGT (Fig. 5 and Supplementary Table S8). Our prediction identified several cross-phyla events within Bacteria (19%) and a few cross-domain events between Archaea and Bacteria (2%), involving functions related to nucleotide repair, chaperones, HSP, antioxidants, and osmoprotectants. The recipient symbionts of these distantly related donors included several species of Proteobacteria, the low salinity nitrite-oxidizing bacteria (LS-NOB) group (phylum Nitrospinota), and *Candidatus Nitrosopumilus* (phylum Nitrososphaerota) (Fig. 5). Conversely, the unassigned donor taxa involved in most of the HGT may be closely related to the recipient taxa (at the genus or species level), possibly reflecting the common close-distance HGT counterpart. The frequency or proportion of HT genes was mostly related to the gene content of each function for cold adaptation, indicating a higher presence of nucleotide repair and fewer osmoprotectants-related annotations across recipient symbionts.

When specifically analyzing HGT events from MAGs of seawater free-living microorganisms to MAGs of sponge symbionts, we observed few HT genes overall, mainly related to transcription and ATM functions (see Supplementary Fig. S6). Importantly, no HT genes involved in cold adaptation were detected in these transfers.

### Cold shock proteins and antioxidants are restricted and acquired by specific bacterial groups and Antarctic sponge species

**Fig. 6:**
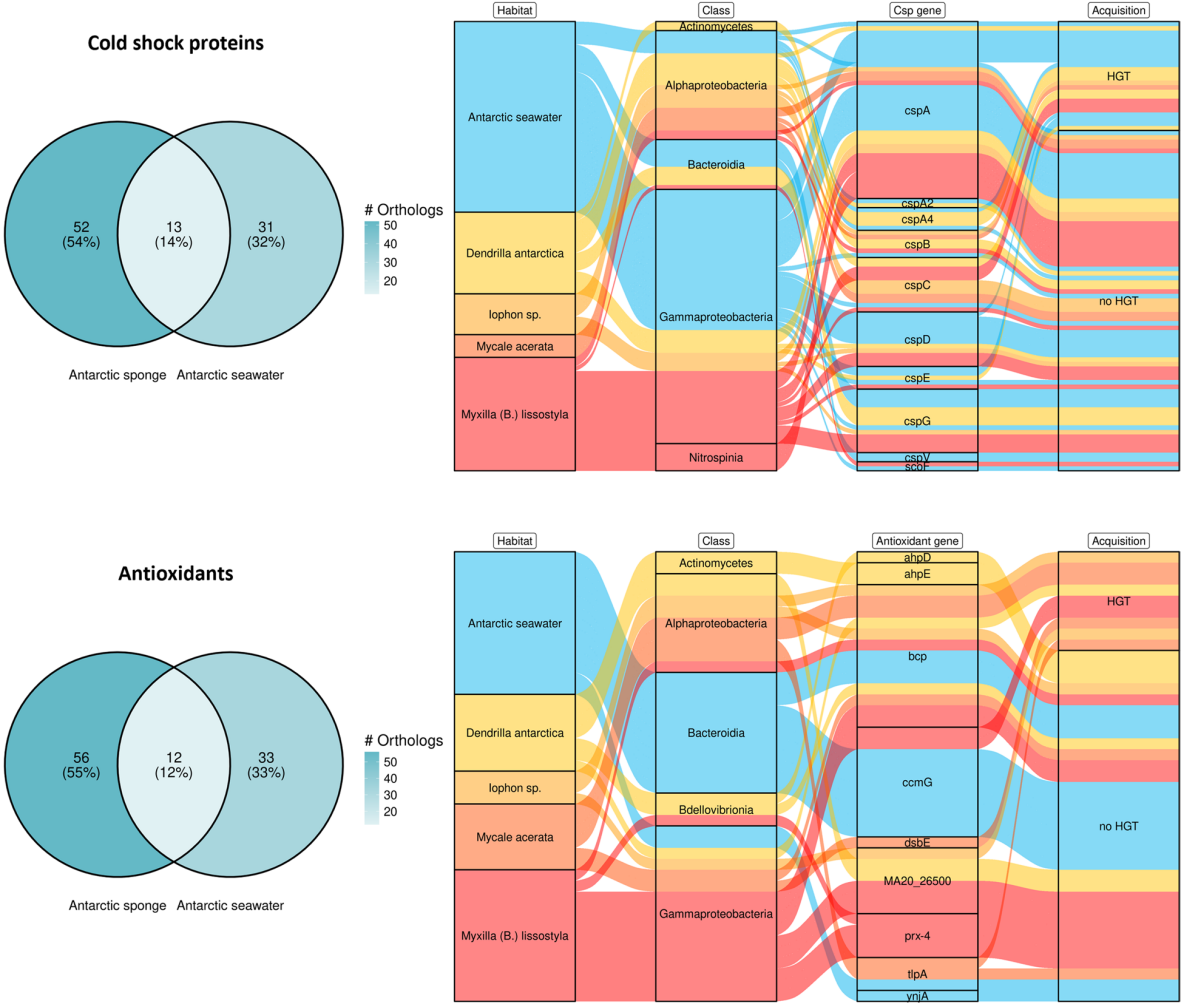
Distribution, proportions, and acquisition of cold shock proteins (Csp) and antioxidants in the microbial classes across sponge species and seawater. Exclusive and shared genes in MAGs (left panels). Distribution and acquisition of well-characterized genes (i.e., with standard nomenclature) in microbial classes across sponge species and seawater (right panels)

We also aimed to focus on the proportion, distribution, and HT genes encoding for members of the cold-shock family of proteins and antioxidants, as they contribute to cold adaptation, presenting an “Antarctic sponge microbiome signature” and a “sponge microbiome signature,” respectively. More than half of the Csp and antioxidant genes were exclusive to Antarctic sponge symbionts, while slightly over 10% were shared with the seawater free-living microorganisms (Fig. 6**,** left panels). When considering only well-characterized genes, these functions seemed to be restricted to specific bacterial groups and sponge species (Fig. 6**,** right panels).

We found Csp in the Gamma- and Alphaproteobacteria, Bacteroidia, Nitrospinia, and Actinomycetes associated with Antarctic sponges. The genes *csp*B and *csp*C were the only ones present in all Antarctic sponge species, although restricted to Proteobacteria, and appeared in a single bacterial MAG from seawater (Fig. 6**,** right upper panel). We found that 5 out of 11 *cspC* orthologs (i.e., gene representatives across Gamma- and Alphaproteobacterial species) and 3 out of 5 *cspB* orthologs from alphaproteobacterial species were putatively acquired via HGT exclusively for the symbiotic lifestyle within the sponges but not for the free-living lifestyle in the seawater. Other characterized Csp presented at least some orthologs HT in free-living bacteria.

For genes identified as antioxidants, we found gene orthologs in Gamma- and Alphaproteobacteria, Bdellovibrionia, and Actinomycetes associated with Antarctic sponges (Fig. 6**,** bottom right panel). Some antioxidants were exclusive to a single sponge species and absent in free-living bacteria. The *ahpD* and *ahpE* genes, encoding for the alkyl hydroperoxide reductase, were found only in sponge symbionts associated with *D. antarctica.* The *dsbE* gene, encoding for the thiol-disulfide interchange protein involved in disulfide bond formation and indirectly aiding in managing oxidative stress, was unique to *Mycale acerata.* Similarly, the gene *prx4*, coding for the peroxiredoxin IV, was identified only in *Myxilla (Burtonanchora) lissostyla*, indicating sponge-species specificity for some antioxidant-encoding genes. Notably, HT antioxidant genes were observed solely in sponge symbionts.

## Discussion

### Exploring functional similarities and differences in Antarctic sponge microbiomes

Previous functional studies on sponge microbiomes from different environments have been described locally and independently. Here, we compared functional marine sponge microbiomes across tropical, temperate, and Antarctic environments. We concentrated on the functions that differentiate Antarctic sponge symbionts and HGT as a mechanism facilitating adaptation and resilience to their specific environment and lifestyle.

Community composition in sponge microbiomes has evidenced remarkable differences between Antarctica and other environments, revealing habitat-specific bacteria [11, 30]. Here, global functional profiles in sponge microbiomes were not characteristic of each environment and exhibited enrichment of typical functional categories. Sponge microbiomes predominantly share a wide range of core functional genes, linked to nutrient cycling, symbiotic lifestyle, and stress resistance, regardless of their taxonomic composition, host phylogenetic diversity, or geographic location [33, 34, 81]. However, we observed that these microbiomes were rich in genes exclusive to each environment, likely encoding equivalent functions. Functional similarity between sponge microbiomes, independent of host or environmental factors, has been noted before [33, 81, 82], as is also the case when comparing the microbiome of marine sponges with the freshwater sponge *Ephydatia muelleri* [83]. Functional equivalence and redundancy likely maintain ecological stability, adaptability, and resilience of sponge ecosystems across environments.

A high abundance of genes related to the symbiotic lifestyle typically characterizes the sponge microbiome, extending beyond geographic locations [33–35, 84]. Our results indicate that Antarctic sponge microbiomes stood out by the number of genes encoding functions for cold adaptation compared to their surrounding seawater and sponges of other environments. However, among these cold adaptation functions, antioxidants exhibited a sponge microbiome signature, while Csp represented a relevant Antarctic sponge microbiome signature. It should be noted, however, that deep-sea sponges, although from tropical regions, also exhibited elevated values for some of these functions, indicating that such high functional representation may extend to cold waters or deep-sea habitats beyond high latitudes.

There are several possible scenarios to explain these signatures:

(i) Antarctic sponge habitat-specific bacteria likely exhibit various unique orthologous gene families (i.e., groups with representatives present exclusively in this environment or as single orthologs due to the absence of specific bacteria elsewhere) that have evolved faster due to the selective pressures of the sponge environment compared to seawater. At the same time, dispersal constraints restrict the horizontal acquisition of Antarctic cold-adapted microorganisms and their genes in tropical and temperate sponges.
(ii) Many orthologous gene families were likely lost in free-living microorganisms closer to the tropics or when they established a symbiotic lifestyle with sponges, since they were no longer essential under warmer temperatures, prioritizing others. Cold and stable conditions at depth may favor the retention of these functions, as observed for deep-sea tropical sponges. The low temperature and biogeography of Antarctica likely play a role in shaping the distinctive sponge microbiome composition previously observed [11, 30] and the cold-related functional differences presented here. However, the abundance of essential metabolic and resistance genes does not vary much between sponge microbiomes across environments and is particularly elevated in the Antarctic seawater microbiome. Due to their free-living lifestyle and ubiquity, microorganisms in seawater often exhibit metabolic versatility that enables them to utilize a broad range of organic and inorganic compounds for survival [85–88]. In contrast, symbionts typically show specialization tailored to the host’s needs rather than exploiting diverse metabolic pathways, especially for obligate and syntrophic members of microbial communities [85, 89].

The abundances of genes related to metal and antibiotic resistance and metabolism (MAR) were higher in Antarctic seawater microbiomes than in sponge microbiomes. A genetic association between metal resistance and antibiotic resistance genes has been proposed through models of co- and cross-selection [90–92]. Seawater can be seen as a reservoir of MAR genes, often hosted on MGE such as plasmids and integrons [90–94]. Previous studies have reported antibiotic and heavy metal resistance in bacterial isolates from Antarctic seawater, particularly in areas with high anthropogenic activity, like King George Island in the Western Antarctic Peninsula [95, 96]. This may explain the strong selection for resistance genes observed in Antarctic seawater microbiomes.

### Role of horizontal gene transfer in shaping metabolism and cold adaptation of Antarctic sponge symbionts

Gamma- and Alphaproteobacteria, known as dominant bacteria across habitats [97–100], stood out by their numerous HT genes compared to other taxa. These sponge symbionts and Bacteroidia significantly surpassed the putative HGT number in seawater free-living counterparts. Specifically, proteobacterial symbionts exceeded the range reported in most bacterial genomes (10–20% of the protein-coding genes) [37]. Most of these genes are involved in lipid, amino acid, and carbon transport and metabolism. HGT has been proposed to facilitate microbial evolutionary adaptation within sponges [101]. We suggest that HGT facilitated the adaptation of some symbionts to changing nutrient conditions in the Antarctic sponge ecosystem over time, providing new metabolic genes or genes that may have been lost due to obligate symbiosis in successive generations. Symbionts with gene loss by streamlining or gene gain by HGT may participate in syntrophic interactions, so not all symbionts need to possess the same capabilities [101]. Also, symbionts can access some nutrients from the host [102].

Here, we measured the HGT contribution to acquiring functions for cold adaptation in Antarctic sponge symbionts by comparing their proportions with (i) functions of known high HGT rate and (ii) free-living microorganisms of the surrounding seawater. The contribution of HGT to cold adaptation was significant, as it displayed proportions similar to the metabolic and resistance functions analyzed in Gamma- and Alphaproteobacteria and Bacteroidia. In contrast, in seawater, HGT contributed less to cold adaptation than to other functions, such as MAR. We hypothesized that HGT related to cold adaptation is primarily ancient, considering that cold Antarctic conditions emerged millions of years ago [103]. The HGT of cold-adaptive functions likely first occurred among seawater microorganisms, with some of them being selected as symbionts by Antarctic sponges, either in the past or currently. Additionally, HGT could serve as a mechanism for distributing core functions among specific symbionts as they associate with and adapt to the sponge environment [33, 101]. Our results indicate that several important metabolic and cold-adaptive genes are part of the Antarctic sponge microbiome functional core, having been horizontally transferred, either recently or in the past, in some current Antarctic sponge symbionts.

It is known that HGT occurrence and frequency vary significantly depending on several factors, including phylogenetic relatedness, ecological niche, and functional gene categories [104–107]. HGT is more likely to occur among closely related microorganisms or among distantly related ones that share the same environment or ecological niche [104, 105], as seen in a microbiome scenario. Here, we identified potential long-distance HGT events related to cold adaptation across distant taxa. Bacteroidia species were frequent donors, while Proteobacteria species were the most frequent recipients. However, the taxonomy of these donors must still be approached with caution, since the HGTector2 software utilized here appears to be more effective at detecting long-distance HGT events rather than close-distance transfers [78].

Nevertheless, a recent study demonstrated that at least 8% of the reference bacterial species from seawater, gut, and soil have experienced cross-phyla HGT within the past 1000 years, revealing that long-distance HGT is more frequent than previously thought [106]. Our results indicated a high percentage of HT genes between bacterial species from different phyla in Antarctic sponges. This finding suggests that these taxa may exhibit high functional compatibility of conjugation machinery and have available integration sites for successful gene integration [108, 109].

Furthermore, our results showed that HGT is more likely to occur between sponge symbionts than between free-living bacteria. This is likely due to the close spatial proximity and higher microbial density in the sponge microenvironment (primarily the mesohyl), which favors gene exchange such as conjugation [32, 110]. These habitat-level differences also explain the relatively few HGT events detected from the free-living seawater bacteria to the sponge symbionts, which likely represent the pre-symbiotic (free-living) phase of horizontally recruited symbionts or scarce ongoing interactions in the sponge environment. These observations align with previous evidence indicating that HGT among gut bacteria is more common than among marine bacteria; likewise, it is more common among bacteria from the same habitat than among bacteria from different habitats [106].

Previous research has indicated that sponge microbiomes are rich in MGE [33, 34]. Our results indicate that ICE is a common feature of sponge microbiomes across environments in contrast to their lower prevalence in Antarctic seawater. Also, all symbiont taxa in Antarctic sponges exhibited higher values of HT ICE than the free-living bacteria from seawater. Therefore, we suggest that conjugation involving ICE serves as a key HGT mechanism for symbiotic adaptation in sponge microbiomes. In contrast, antibiotic and metal resistance enrichment in Antarctic seawater microbiomes may predominantly be acquired through other MGE, such as plasmids. Distinct evolutionary pressures and ecological roles in sponges and seawater could explain these differences. For instance, ICE allows for more stable integration [111], which favors the vertical transmission of specialized functions related to nutrient host requirements in sponge symbionts [35]. On the other hand, plasmids enable rapid and transient adaptation to environmental stressors [111], which is more critical for free-living microorganisms in seawater [95]. Moreover, ICE are more frequently transferred among distant taxa than plasmids, as they integrate into the chromosome but show less frequent gene exchange [112].

Csp and antioxidants, which primarily differentiated the functional Antarctic sponge microbiome from its surrounding environment, also exhibited a restrictive distribution to specific bacterial groups and sponge species. This pattern is likely an example that not all sponge symbionts must perform the same functional niche concerning cold adaptation, as reported for nutritional specialization in symbionts associated with the Mediterranean sponge *Aplysina aerophoba* [113]. Additionally, diverse members of protein families CspA, CspB, and CspC and their orthologs distributed across different bacterial groups provide equivalent functions within Antarctic sponge microbiomes. This way, these functions for cold adaptation can be conserved, even though the sponge species hosts specific bacterial groups.

Csp act like mRNA chaperones, preventing the formation of secondary structures during transcription and translation, hindered at low temperatures [41, 114]. Here, we show that c*sp*C and *csp*B are genes for cold adaptation that potentially undergo HGT only in sponge symbionts, being also present in all the sponge species analyzed. CspC is a known effector of the Secretion System Type IV [115], a complex responsible for exporting DNA and proteins across the cell membranes of bacteria and archaea and enabling conjugation [116, 117]. However, further research is needed to determine the role of important genes related to cold adaptation, such as cspC, in host-symbiont interactions, which may help explain the Antarctic sponge microbiome signature observed here.

As we hypothesize that HGT for cold adaptation is primarily ancient, we also propose that HGT for metabolic and symbiotic functions may be primarily recent: environmentally acquired microorganisms (facultative symbionts) may act mainly as potential donors of metabolic genes to vertically transmitted microorganisms (commonly obligate symbionts). In contrast, these latter may act as donors of symbiotic genes to recipient facultative ones.

Future studies should focus on up- and downregulated genes for cold adaptation in Antarctic sponge symbionts compared to those in sponges from other environments. While host phylogeny can influence microbial community composition, functional profiles in sponge microbiomes often show high conservation across lineages; thus, cross-taxon comparisons should be interpreted with due caution. Furthermore, variability in DNA or RNA extraction protocols across studies may still represent a potential limitation when comparing multiple datasets or substrates. Also, MAG-based analyses are still biased in retrieving more abundant taxa from microbiomes. Accordingly, further metagenomic and culture-based methods are expected to reveal more HGT events and HT genes in the microbiomes of Polar sponges. Moreover, further exploration of the active transcription of HGT-acquired genes can demonstrate their successful integration into the recipient genomes.

## Conclusions

Antarctic sponge microbiomes are enriched in genes linked to cold adaptation, especially compared to their surrounding seawater and sponges from other environments. Selective pressures of the Antarctic sponge environment, their dispersal constraints, and gene loss in sponges inhabiting warmer waters likely influence the functional composition and characteristics of the Antarctic sponge microbiome observed here, although host phylogeny may introduce subtle variability in comparisons. HGT, primarily driven by ICE, seems to serve as a primary mechanism for functional differentiation in Antarctic sponge symbionts, contributing similarly to cold adaptation as other metabolic functions, with a strong signal in Gamma- and Alphaproteobacteria and Bacteroidia. Overall, our findings highlight the functional and evolutionary signatures that underpin microbial resilience and symbiosis in polar benthic environments. This study provides a foundational step toward understanding the functional signatures and evolutionary mechanisms driving microbial adaptation within Antarctic sponge microbiomes.

## Supplementary Information

Additional file 1: Supplementary Figure S1. Supplementary Figure S1. Exclusive and shared genes annotated between Antarctic, tropical, and temperate sponge microbiomes. Supplementary Figure S2. Presence/absence of genes encoding functions for cold adaptation in the microbiomes of Antarctic, tropical, and temperate sponges and Antarctic seawater. The metagenome sizes of all samples were normalized to the sample with the smallest size. The Ward method was used to perform clustering. Supplementary Figure S3. Percentage of genes encoding functions related to cold adaptation and metabolism non-significantly higher in Antarctic sponge microbiomes compared to their surrounding seawater and temperate and tropical sponge microbiomes. ****: *p* < 0.0001, ***: *p* < 0.001, **: *p* < 0.01,*: p < 0.05. ATM: Amino acid transport and metabolism, CTM: Carbohydrate transport and metabolism, EPC: Energy production and conversion, LTM: Lipid transport and metabolism, MAR: Metal and antibiotic resistance and metabolism. Supplementary Figure S4. Exclusive and shared *csp* genes between Antarctic, tropical, and temperate sponge microbiomes. Supplementary Figure S5. Association between the number of genes annotated and the number of horizontally transferred (HT) genes in each functional group based on different calculations: (A) The number of HT genes within each functional group, relative to the total number of HT genes. (B) The number of HT genes per total gene content within each functional group. Pearson was used to test correlations between the variables. ATM: Amino acid transport and metabolism, CTM: Carbohydrate transport and metabolism, EPC: Energy production and conversion, LTM: Lipid transport and metabolism, MAR: Metal and antibiotic resistance and metabolism, ICE: Machinery of Integrative and conjugative elements. Supplementary Figure S6. Putative genes horizontally acquired in MAGs of Antarctic sponge symbionts from MAGs of free-living bacteria in the surrounding seawater. The X-axis displays the sponge species (above) and the taxonomy of the recipient MAGs (below).

Additional file 2: Supplementary Table S1. Supplementary Table S1. Metadata associated with the Antarctic, tropical, and temperate sponge microbiomes and Antarctic seawater microbiomes used in this study. Asterisks in "Sponge_status" indicate apparent classification of LMA sponges based on alpha diversity and phylum composition. Supplementary Table S2. Quantity and quality parameters of metagenomes (contigs, genes, annotations). Supplementary Table S3. List and counts of non-redundant genes classified into cold adaptation functions across all analyzed metagenomes. Supplementary Table S4. List and counts of non-redundant genes classified into the analyzed metabolic, resistance, and conjugative functions across all analyzed metagenomes. Supplementary Table S5. Pairwise PERMANOVA comparisons for the effect of the environment on the gene composition of microbiomes based on Bray–Curtis dissimilarities. Significance: "." indicates *p* < 0.05, "*" indicates *p* < 0.01, "**" indicates *p* ≤ 0.001. Supplementary Table S6. Gene proportions (%), abundance (RPKG), and diversity (Shannon index) of the functional groups analyzed in this study. The functional groups comprise cold adaptation, amino acid transport and metabolism (ATM), carbohydrate transport and metabolism (CTM), energy production and conversion (EPC), lipid transport and metabolism (LTM), metal and antibiotic resistance and metabolism (MAR), and machinery of integrative and conjugative elements (ICE). Supplementary Table S7. Taxonomy-assignment and quantity and quality parameters of MAGs (contigs, genes, annotations). Supplementary Table S8. Donor and recipient taxa of horizontally transferred functions for cold adaptation and the type of HGT event.

## Data Availability

Metagenome raw paired-end reads from Antarctic sponges and seawater generated in this study are available in the SRA repository of NCBI under the BioProject accession number PRJNA1263899 (https://www.ncbi.nlm.nih.gov/bioproject/PRJNA1263899). Accession numbers of the equivalent metagenome reads from tropical and temperate sponges analyzed during this study and metadata are included in this article (Additional Material 2, Supplementary Table S1). MAGs from Antarctic sponges and seawater are available under the same BioProject number PRJNA1263899 (https://www.ncbi.nlm.nih.gov/bioproject/PRJNA1263899). The scripts/code used for the data processing and analysis are available in the GitHub repository: https://github.com/mdelacuba/script-repo-2025.
