# Supplementary figures and images for: Cold adaptation and horizontal gene transfer shape Antarctic sponge microbiomes
Source: Microbiome. 2025 Nov 26;13:243. doi: 10.1186/s40168-025-02262-z (PMC12659273; doi:10.1186/s40168-025-02262-z)

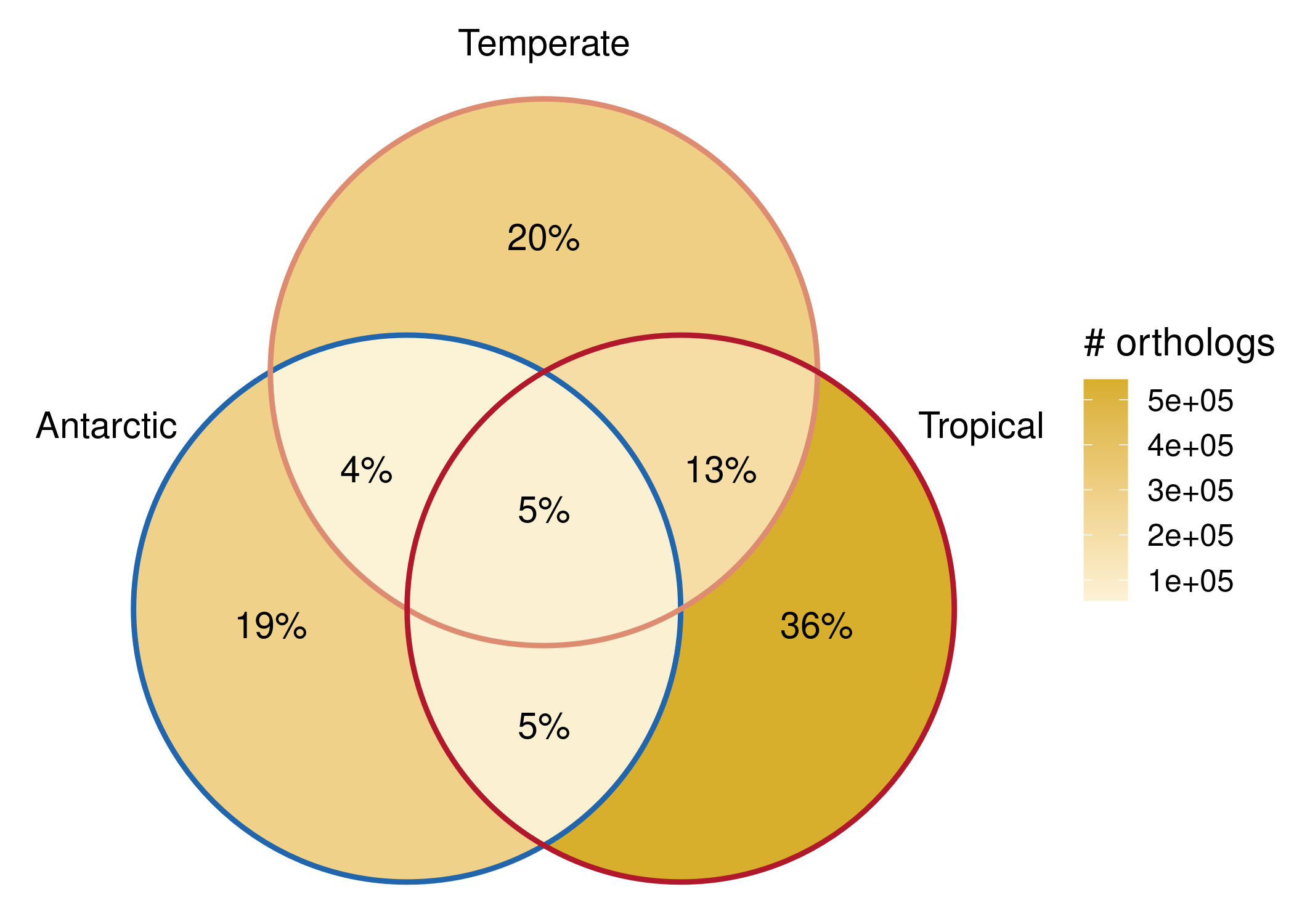

Supplement: Supplementary file 2 — Additional file 1: Supplementary Figure S1. Supplementary Figure S1. Exclusive and shared genes annotated between Antarctic, tropical, and temperate sponge microbiomes. Supplementary Figure S2. Presence/absence of genes encoding functions for cold adaptation in the microbiomes of Antarctic, tropical, and temperate sponges and Antarctic seawater. The metagenome sizes of all samples were normalized to the sample with the smallest size. The Ward method was used to perform clustering. Supplementary Figure S3. Percentage of genes encoding functions related to cold adaptation and metabolism non-significantly higher in Antarctic sponge microbiomes compared to their surrounding seawater and temperate and tropical sponge microbiomes. ****: p < 0.0001, ***: p < 0.001, **: p < 0.01,*: p < 0.05. ATM: Amino acid transport and metabolism, CTM: Carbohydrate transport and metabolism, EPC: Energy production and conversion, LTM: Lipid transport and metabolism, MAR: Metal and antibiotic resistance and metabolism. Supplementary Figure S4. Exclusive and shared csp genes between Antarctic, tropical, and temperate sponge microbiomes. Supplementary Figure S5. Association between the number of genes annotated and the number of horizontally transferred (HT) genes in each functional group based on different calculations: (A) The number of HT genes within each functional group, relative to the total number of HT genes. (B) The number of HT genes per total gene content within each functional group. Pearson was used to test correlations between the variables. ATM: Amino acid transport and metabolism, CTM: Carbohydrate transport and metabolism, EPC: Energy production and conversion, LTM: Lipid transport and metabolism, MAR: Metal and antibiotic resistance and metabolism, ICE: Machinery of Integrative and conjugative elements. Supplementary Figure S6. Putative genes horizontally acquired in MAGs of Antarctic sponge symbionts from MAGs of free-living bacteria in the surrounding seawater. The X-axis [file 40168_2025_2262_MOESM1_ESM.zip › FigureS1.png]

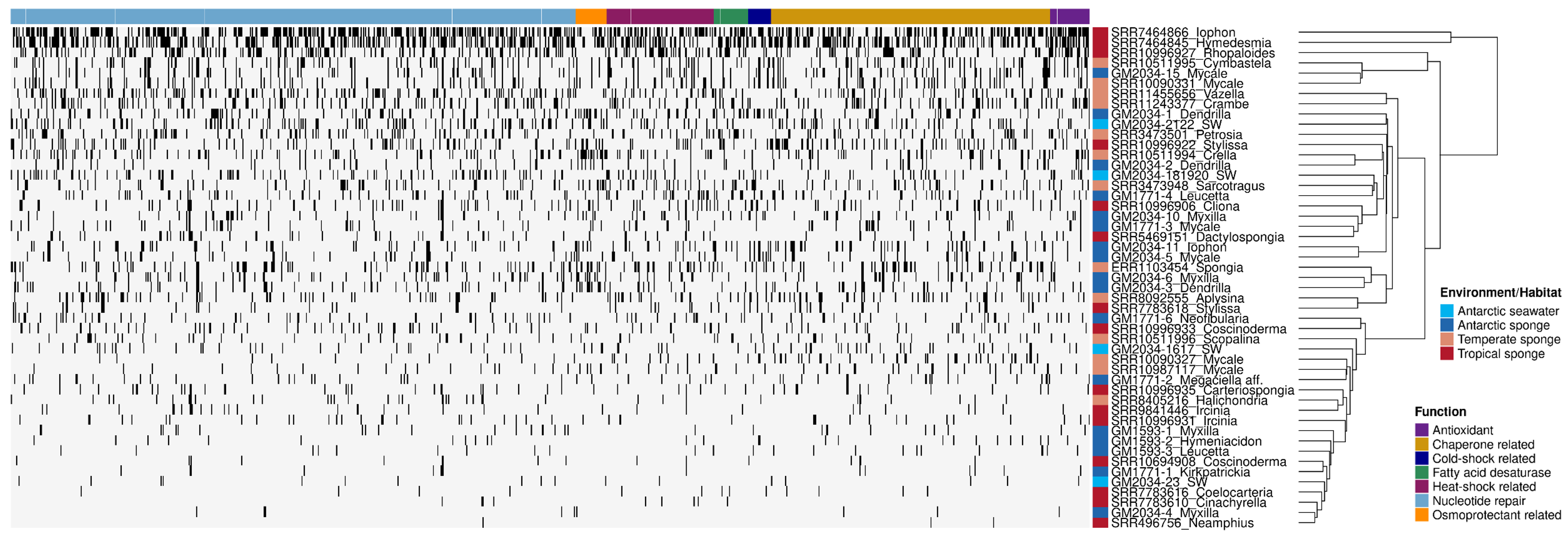

Supplement: Supplementary file 2 — Additional file 1: Supplementary Figure S1. Supplementary Figure S1. Exclusive and shared genes annotated between Antarctic, tropical, and temperate sponge microbiomes. Supplementary Figure S2. Presence/absence of genes encoding functions for cold adaptation in the microbiomes of Antarctic, tropical, and temperate sponges and Antarctic seawater. The metagenome sizes of all samples were normalized to the sample with the smallest size. The Ward method was used to perform clustering. Supplementary Figure S3. Percentage of genes encoding functions related to cold adaptation and metabolism non-significantly higher in Antarctic sponge microbiomes compared to their surrounding seawater and temperate and tropical sponge microbiomes. ****: p < 0.0001, ***: p < 0.001, **: p < 0.01,*: p < 0.05. ATM: Amino acid transport and metabolism, CTM: Carbohydrate transport and metabolism, EPC: Energy production and conversion, LTM: Lipid transport and metabolism, MAR: Metal and antibiotic resistance and metabolism. Supplementary Figure S4. Exclusive and shared csp genes between Antarctic, tropical, and temperate sponge microbiomes. Supplementary Figure S5. Association between the number of genes annotated and the number of horizontally transferred (HT) genes in each functional group based on different calculations: (A) The number of HT genes within each functional group, relative to the total number of HT genes. (B) The number of HT genes per total gene content within each functional group. Pearson was used to test correlations between the variables. ATM: Amino acid transport and metabolism, CTM: Carbohydrate transport and metabolism, EPC: Energy production and conversion, LTM: Lipid transport and metabolism, MAR: Metal and antibiotic resistance and metabolism, ICE: Machinery of Integrative and conjugative elements. Supplementary Figure S6. Putative genes horizontally acquired in MAGs of Antarctic sponge symbionts from MAGs of free-living bacteria in the surrounding seawater. The X-axis [file 40168_2025_2262_MOESM1_ESM.zip › FigureS2.png]

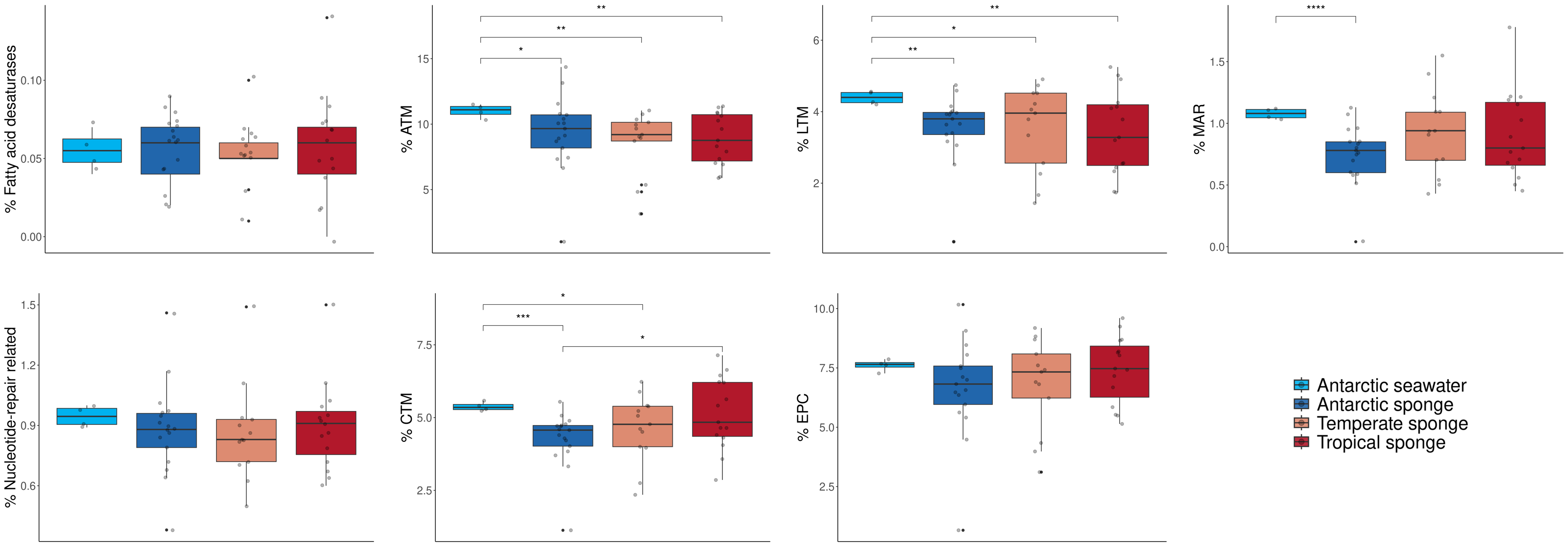

Supplement: Supplementary file 2 — Additional file 1: Supplementary Figure S1. Supplementary Figure S1. Exclusive and shared genes annotated between Antarctic, tropical, and temperate sponge microbiomes. Supplementary Figure S2. Presence/absence of genes encoding functions for cold adaptation in the microbiomes of Antarctic, tropical, and temperate sponges and Antarctic seawater. The metagenome sizes of all samples were normalized to the sample with the smallest size. The Ward method was used to perform clustering. Supplementary Figure S3. Percentage of genes encoding functions related to cold adaptation and metabolism non-significantly higher in Antarctic sponge microbiomes compared to their surrounding seawater and temperate and tropical sponge microbiomes. ****: p < 0.0001, ***: p < 0.001, **: p < 0.01,*: p < 0.05. ATM: Amino acid transport and metabolism, CTM: Carbohydrate transport and metabolism, EPC: Energy production and conversion, LTM: Lipid transport and metabolism, MAR: Metal and antibiotic resistance and metabolism. Supplementary Figure S4. Exclusive and shared csp genes between Antarctic, tropical, and temperate sponge microbiomes. Supplementary Figure S5. Association between the number of genes annotated and the number of horizontally transferred (HT) genes in each functional group based on different calculations: (A) The number of HT genes within each functional group, relative to the total number of HT genes. (B) The number of HT genes per total gene content within each functional group. Pearson was used to test correlations between the variables. ATM: Amino acid transport and metabolism, CTM: Carbohydrate transport and metabolism, EPC: Energy production and conversion, LTM: Lipid transport and metabolism, MAR: Metal and antibiotic resistance and metabolism, ICE: Machinery of Integrative and conjugative elements. Supplementary Figure S6. Putative genes horizontally acquired in MAGs of Antarctic sponge symbionts from MAGs of free-living bacteria in the surrounding seawater. The X-axis [file 40168_2025_2262_MOESM1_ESM.zip › FigureS3.png]

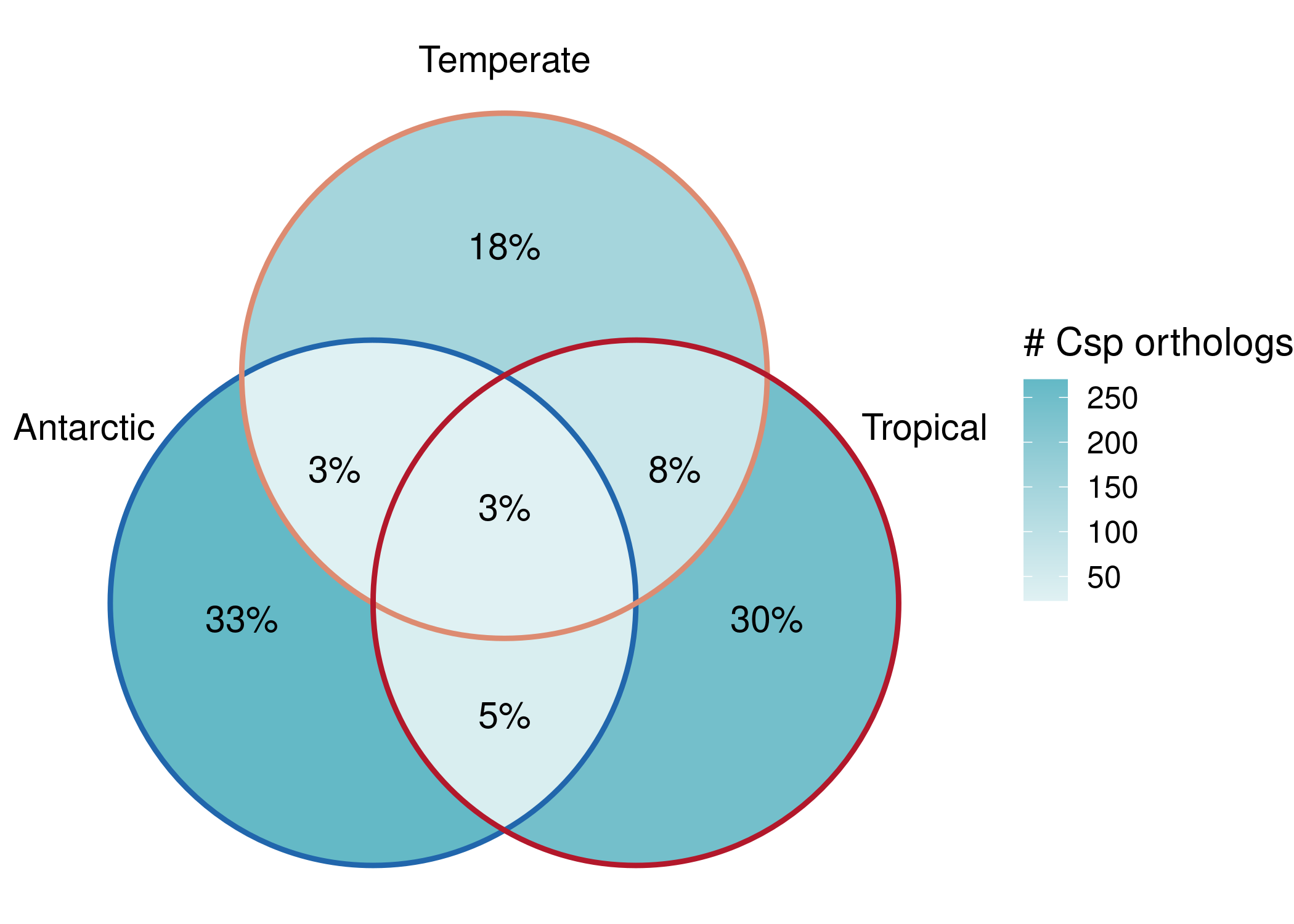

Supplement: Supplementary file 2 — Additional file 1: Supplementary Figure S1. Supplementary Figure S1. Exclusive and shared genes annotated between Antarctic, tropical, and temperate sponge microbiomes. Supplementary Figure S2. Presence/absence of genes encoding functions for cold adaptation in the microbiomes of Antarctic, tropical, and temperate sponges and Antarctic seawater. The metagenome sizes of all samples were normalized to the sample with the smallest size. The Ward method was used to perform clustering. Supplementary Figure S3. Percentage of genes encoding functions related to cold adaptation and metabolism non-significantly higher in Antarctic sponge microbiomes compared to their surrounding seawater and temperate and tropical sponge microbiomes. ****: p < 0.0001, ***: p < 0.001, **: p < 0.01,*: p < 0.05. ATM: Amino acid transport and metabolism, CTM: Carbohydrate transport and metabolism, EPC: Energy production and conversion, LTM: Lipid transport and metabolism, MAR: Metal and antibiotic resistance and metabolism. Supplementary Figure S4. Exclusive and shared csp genes between Antarctic, tropical, and temperate sponge microbiomes. Supplementary Figure S5. Association between the number of genes annotated and the number of horizontally transferred (HT) genes in each functional group based on different calculations: (A) The number of HT genes within each functional group, relative to the total number of HT genes. (B) The number of HT genes per total gene content within each functional group. Pearson was used to test correlations between the variables. ATM: Amino acid transport and metabolism, CTM: Carbohydrate transport and metabolism, EPC: Energy production and conversion, LTM: Lipid transport and metabolism, MAR: Metal and antibiotic resistance and metabolism, ICE: Machinery of Integrative and conjugative elements. Supplementary Figure S6. Putative genes horizontally acquired in MAGs of Antarctic sponge symbionts from MAGs of free-living bacteria in the surrounding seawater. The X-axis [file 40168_2025_2262_MOESM1_ESM.zip › FigureS4.png]

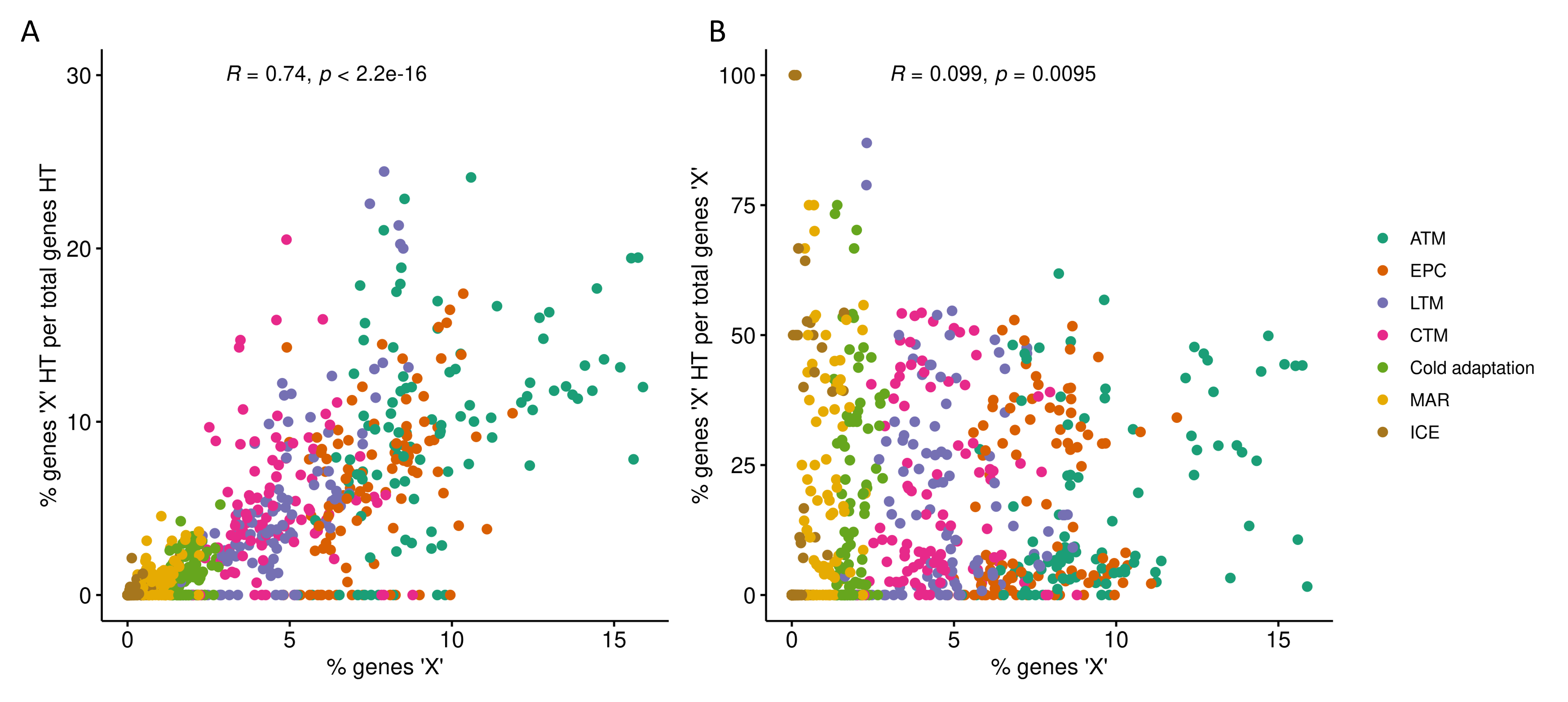

Supplement: Supplementary file 2 — Additional file 1: Supplementary Figure S1. Supplementary Figure S1. Exclusive and shared genes annotated between Antarctic, tropical, and temperate sponge microbiomes. Supplementary Figure S2. Presence/absence of genes encoding functions for cold adaptation in the microbiomes of Antarctic, tropical, and temperate sponges and Antarctic seawater. The metagenome sizes of all samples were normalized to the sample with the smallest size. The Ward method was used to perform clustering. Supplementary Figure S3. Percentage of genes encoding functions related to cold adaptation and metabolism non-significantly higher in Antarctic sponge microbiomes compared to their surrounding seawater and temperate and tropical sponge microbiomes. ****: p < 0.0001, ***: p < 0.001, **: p < 0.01,*: p < 0.05. ATM: Amino acid transport and metabolism, CTM: Carbohydrate transport and metabolism, EPC: Energy production and conversion, LTM: Lipid transport and metabolism, MAR: Metal and antibiotic resistance and metabolism. Supplementary Figure S4. Exclusive and shared csp genes between Antarctic, tropical, and temperate sponge microbiomes. Supplementary Figure S5. Association between the number of genes annotated and the number of horizontally transferred (HT) genes in each functional group based on different calculations: (A) The number of HT genes within each functional group, relative to the total number of HT genes. (B) The number of HT genes per total gene content within each functional group. Pearson was used to test correlations between the variables. ATM: Amino acid transport and metabolism, CTM: Carbohydrate transport and metabolism, EPC: Energy production and conversion, LTM: Lipid transport and metabolism, MAR: Metal and antibiotic resistance and metabolism, ICE: Machinery of Integrative and conjugative elements. Supplementary Figure S6. Putative genes horizontally acquired in MAGs of Antarctic sponge symbionts from MAGs of free-living bacteria in the surrounding seawater. The X-axis [file 40168_2025_2262_MOESM1_ESM.zip › FigureS5.png]

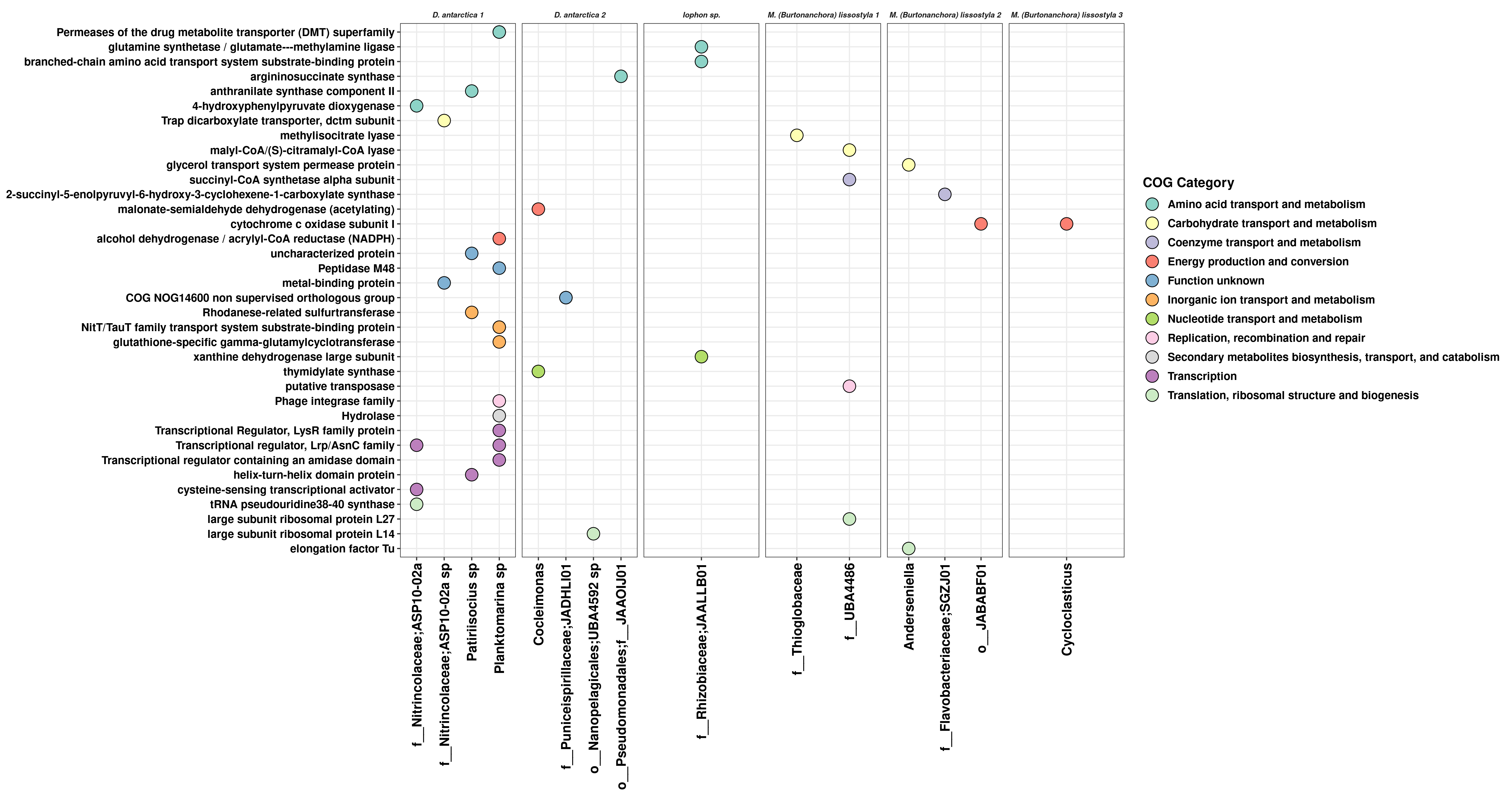

Supplement: Supplementary file 2 — Additional file 1: Supplementary Figure S1. Supplementary Figure S1. Exclusive and shared genes annotated between Antarctic, tropical, and temperate sponge microbiomes. Supplementary Figure S2. Presence/absence of genes encoding functions for cold adaptation in the microbiomes of Antarctic, tropical, and temperate sponges and Antarctic seawater. The metagenome sizes of all samples were normalized to the sample with the smallest size. The Ward method was used to perform clustering. Supplementary Figure S3. Percentage of genes encoding functions related to cold adaptation and metabolism non-significantly higher in Antarctic sponge microbiomes compared to their surrounding seawater and temperate and tropical sponge microbiomes. ****: p < 0.0001, ***: p < 0.001, **: p < 0.01,*: p < 0.05. ATM: Amino acid transport and metabolism, CTM: Carbohydrate transport and metabolism, EPC: Energy production and conversion, LTM: Lipid transport and metabolism, MAR: Metal and antibiotic resistance and metabolism. Supplementary Figure S4. Exclusive and shared csp genes between Antarctic, tropical, and temperate sponge microbiomes. Supplementary Figure S5. Association between the number of genes annotated and the number of horizontally transferred (HT) genes in each functional group based on different calculations: (A) The number of HT genes within each functional group, relative to the total number of HT genes. (B) The number of HT genes per total gene content within each functional group. Pearson was used to test correlations between the variables. ATM: Amino acid transport and metabolism, CTM: Carbohydrate transport and metabolism, EPC: Energy production and conversion, LTM: Lipid transport and metabolism, MAR: Metal and antibiotic resistance and metabolism, ICE: Machinery of Integrative and conjugative elements. Supplementary Figure S6. Putative genes horizontally acquired in MAGs of Antarctic sponge symbionts from MAGs of free-living bacteria in the surrounding seawater. The X-axis [file 40168_2025_2262_MOESM1_ESM.zip › FigureS6.png]
